# Siglec-15 is a glyco-immune checkpoint in prostate cancer regulating immune evasion and metastasis

**DOI:** 10.64898/2026.08.07.743480

**Published:** 2026-08-09

**Authors:** Nicoll Matthews, Feier Zeng, Kirsty Hodgson, Matthew Fisher, Ziqian Peng, Libby Blencoe, Margarita Orozco-Moreno, Ella P Dennis, Liancheng Lu, Michelle A Lawson, Shenglin Mei, David B Sykes, Dallas B Flies, Richard Beatson, Ning Wang, Jennifer Munkley

## Abstract

Prostate cancer is a leading cause of cancer-related mortality in men, and effective treatment options are limited for advanced and metastatic disease. The sialoglycan immune checkpoint Siglec-15 has emerged as a key mediator of tumour-associated immune suppression in several malignancies; however, its expression and functional role in prostate cancer remain poorly defined. Here, using dual immunofluorescence and immunohistochemistry, we demonstrate that Siglec-15 is expressed by prostate tumour epithelial cells, immunosuppressive macrophage phenotypes, and bone-resorbing osteoclasts within the tumour microenvironment. Mechanistically, we show that direct Siglec-15 receptor crosslinking, either by antibodies or tumour cell-derived conditioned medium, promotes monocyte-to-macrophage differentiation, generating macrophages with immunosuppressive and pathogenic phenotypes. Using therapeutic antibodies, we show that Siglec-15 blockade suppresses supernatant-induced monocyte to macrophage differentiation, allowing for the recovery of CD8⁺ T-cell activation. Furthermore, we reveal that macrophage colony-stimulating factor (M-CSF) driven monocyte-derived macrophage differentiation is partially dependent on Siglec-15 signalling, with Siglec-15 blockade enhancing CD8⁺ T-cell responses. In addition, anti-Siglec-15 treatment suppressed osteoclast differentiation, highlighting a dual role for Siglec-15 in prostate cancer immune suppression and bone remodelling. Consistent with these *in vitro* findings, therapeutic Siglec-15 blockade significantly reduced subcutaneous tumour growth in a CD8⁺ T-cell-dependent manner and prolonged survival in a mouse model of prostate cancer metastasis. Together, these findings identify Siglec-15 as a central regulator of the prostate cancer glyco-immune axis, linking tumour-associated macrophage immune suppression with osteoclast-mediated bone remodelling, providing a compelling rationale for the clinical development of Siglec-15-targeted therapies for patients with advanced disease.

## Full Text Availability

The license terms selected by the author(s) for this preprint version do not permit archiving in PMC. The full text is available from the preprint server.

